# Speed of intravenous fluid on glycocalyx integrity and central blood volume in a porcine septic shock model

**DOI:** 10.1186/s40635-026-00893-6

**Published:** 2026-04-24

**Authors:** Yosef Levenbrown, Md Jobayer Hossain, Paul T. Fawcett, Marjorie Postell, Anne M. Hesek, Lynell S. Jones, James P. Keith, Thomas H. Shaffer

**Affiliations:** 1 Division of Pediatric Critical Care, Nemours Children’s Health, Delaware, 1600 Rockland Road, Wilmington, DE 19803 USA; 2 Department of Pediatrics, Sidney Kimmel Medical School of Thomas Jefferson University, Philadelphia, PA USA; 3 Nemours Biomedical Research, Wilmington, DE USA; 4 Department of Applied Economics and Statistics, University of Delaware, Newark, DE USA; 5 Department of Respiratory Care, Integrative Medicine, Nemours Children’s Health, Wilmington, DE USA; 6 Nemours Biomedical Research/Research Lung Center, Wilmington, DE USA; 7 Department of Pediatrics, Sidney Kimmel Medical College at Thomas Jefferson University, Philadelphia, PA USA; 8 Department of Pediatrics, Lewis Katz School of Medicine at Temple University, Philadelphia, PA USA

**Keywords:** Sepsis, Endothelium, IV fluid, Resuscitation, Shock

## Abstract

**Background:**

It is unclear whether the rate of intravenous fluid (IVF) administration in sepsis influences glycocalyx breakdown. This study aimed to measure the effect of rapid versus slow fluid bolus in a septic pig model on the integrity of the glycocalyx by measuring the serum levels of heparan sulfate and syndecan-1, breakdown products of the glycocalyx. Because endothelial glycocalyx is composed of apical cell membrane-bound proteoglycans such as syndecan-1, covalently bound with long linear glycosaminoglycans such as heparan sulfate, shedding of these different glycocalyx components is closely related. Additionally, we measured central blood volume (blood volume in the heart and lungs) to determine the effect of fluid administration rate on intravascular volume. Plasma membrane type-1 matrix metalloproteinase (MT1-MMP) levels (indicating vascular shear stress) were compared between groups to assess whether fast IVF boluses generate greater endothelial shearing forces than slow boluses.

**Methods:**

A porcine septic shock model using lipopolysaccharide-induced endotoxemia was used. We randomized 22 piglets to receive two fluid boluses of 20 mL/kg each 30 min apart; 11 received the fluid bolus over 5 min and 11 over 20 min. We monitored the central blood volume index (CBVI) using ultrasound dilution. Measurements of glycocalyx degradation markers, heparan sulfate and syndecan-1, MT1-MMP, and CBVI were obtained at the onset of septic shock and every 30 min for four measurements. Study variables were summarized as mean (standard deviation) by fast versus slow IVF groups across measurement time points. To assess the mean trends in outcomes (syndecan-1, heparan sulfate, MT1-MMP, CBVI) between the two speeds of IVF administration over time, a linear mixed-effects model with a first-order autoregressive within-subject correlation structure was applied.

**Results:**

Serum heparan sulfate and syndecan-1 levels showed no significant differences (*P* = 0.405 and 0.571, respectively). Baseline-adjusted mean central blood volumes over the four measurements were greater in the slow IVF versus fast group; however, the difference fell short of statistical significance (*P* = 0.073), indicating a nonsignificant trend toward greater central blood volume with slower infusion. Although MT1-MMP levels increased with time, there was no between-group difference in the MT1-MMP levels.

**Conclusions:**

There was no difference in glycocalyx degradation or vascular shear stress between an IVF bolus given over 5 min compared with 20 min. Central blood volumes trended greater in the slow IVF group compared with the fast IVF group.

## Background

Sepsis is defined as an infection with a dysregulated host response resulting in life-threatening organ dysfunction. Septic shock is sepsis with profound circulatory, cellular, or metabolic alterations associated with substantially higher mortality [1]. The burden of sepsis in both adult and pediatric patients is significant. In adult patients, recent reports estimate that in 2021, 166 million sepsis cases and 21.4 million all-cause sepsis-related deaths occurred globally, representing 31.5% of total global deaths [2]. Epidemiologic studies have found that pediatric sepsis results in 8% of all pediatric intensive care unit admissions and contributes to 25% of pediatric intensive care unit deaths [3, 4]. Globally, there are an estimated 1.2 million cases of childhood sepsis annually [5]. Mortality in children with sepsis ranges from 4% to as high as 50% [6].

Early identification and appropriate resuscitation and management are critical to optimizing outcomes for patients with sepsis. The administration of intravenous fluid (IVF) during sepsis is one of the frontline therapies to replete the severely diminished intravascular fluid volume resulting from capillary leak and vasodilation [6]. It is now appreciated that the effect of IVF in replenishing the intravascular fluid volume in sepsis is transient, as the fluid administered does not remain in the intravascular space for more than an hour [7–10]. Prior studies have demonstrated that rapid administration of IVF compared with slower administration of IVF is associated with more adverse outcomes, including a higher risk of requiring mechanical ventilation and a greater need for vasopressors [11, 12]. These adverse effects seen with faster administration of the IVF and the transient benefit provided by IVF are presumed to be secondary to the extravasation of IVF that occurs with fluid bolus therapy (FBT) in sepsis. Failure to maintain the IVF in the intravascular compartment is likely due, in part, to damage of the endothelial glycocalyx.

The endothelial glycocalyx plays a crucial role in regulating cell movement dynamics in the microcirculation, maintaining vascular tone and permeability, facilitating platelet and coagulation function, and modulating the immune response [13–15]. Sepsis is known to cause degradation of the glycocalyx, resulting in endothelial dysfunction, the central pathology in sepsis and septic shock. Inflammatory cytokines, especially tumor necrosis factor-alpha (TNF-α), have been implicated in the degradation of the glycocalyx in sepsis [16–19]. Secondary causes of glycocalyx degradation in sepsis have been identified. IVF therapy has a damaging effect on the glycocalyx. Both the volume of IVF and the type of IVF (unbalanced solutions more than balanced solutions) are associated with glycocalyx degradation [14, 20, 21], potentially worsening the underlying pathology in sepsis. Given the importance of glycocalyx integrity to vascular homeostasis, exacerbation of sepsis-induced glycocalyx degradation by intravenous fluids would be expected to worsen inflammatory organ injury and microcirculatory dysfunction, significantly impacting patient outcomes.

It has been speculated but not proven that the speed of IVF administration can worsen glycocalyx breakdown. Using a porcine septic shock model, with sepsis induced by lipopolysaccharide from *Escherichia coli* serotype 0111:B4, this study assesses the impact of slow versus fast IVF administration on the breakdown of the endothelial glycocalyx. To test this hypothesis, we measured plasma levels of heparan sulfate and syndecan-1, two breakdown products of the glycocalyx, to determine whether administration of a fast IVF bolus (two fluid boluses of 20 mL/kg given over 5 min) results in greater glycocalyx breakdown compared with a slow IVF bolus (given over 20 min). Additionally, plasma membrane type-1 matrix metalloproteinase (MT1-MMP) levels were measured between the two groups. MT1-MMP has been shown to increase with vascular shearing forces [22] and was therefore measured to determine if a fast IVF bolus results in greater shearing forces on the vascular endothelium compared with a slow IVF bolus. Finally, the central blood volume index (CBVI), which represents the volume of blood in the heart and lungs, was compared between the two groups to determine if fast FBT compared with slow FBT results in decreased circulating blood volume in the central circulation, potentially due to worsening third spacing of the IVF.

## Methods

### Animal preparation

Young Landrace–Yorkshire piglets (*n* = 22) weighing 25–35 kg were used. The pigs were managed in accordance with the NIH guidelines for the care and use of laboratory animals. The pigs were purchased from the same supplier, received by our laboratory on the morning of each study and examined by the staff of the Life Science Center at Nemours Children’s Hospital. The pigs underwent a veterinary health evaluation and certification prior to shipping to the laboratory. The Nemours Institutional Animal Care and Use Committee approved the experimental protocol (Approval number: Rsp22-11015-002).

### Anesthesia and monitoring

The pigs received initial sedation with two intramuscular injections 10 min apart of 1 mL/kg of KAX, an anesthetic cocktail containing ketamine 23 mg/mL, acepromazine 0.58 mg/mL, and xylazine 0.8 mg/mL. Following the initial sedation, arterial (left carotid) and central venous catheters (right internal jugular line) were placed using standard cut-down techniques. The pigs were then intubated via midline tracheostomy using a cuffed endotracheal tube. The animals were ventilated (Servo-I Ventilator, Getinge, Wayne, NJ) using volume control mode ventilation, with a tidal volume of 7 mL/kg, a rate of 25 breaths per minute, positive and expiratory pressure of 5 cm H_2_O, and fraction of inspired oxygen of 1. The ventilator breath rate was adjusted based on the initial arterial blood gas results to maintain a carbon dioxide level in the 35–45 cm H_2_O range. Anesthesia was maintained following these initial procedures with continuous infusions of ketamine 15 mg/kg/h and propofol 6 mg/kg/h. Adequate sedation was confirmed by hoof pinch using a surgical clamp, with none of the animals needing escalation in their anesthesia. This anesthesia protocol has been used by our laboratory for previous studies and has been demonstrated to be effective in establishing an appropriate degree of anesthesia for this study [23, 24].

The COstatus cardiac output monitor (Transonic Systems, Inc., Ithaca, NY) was connected to both the central venous line and the arterial line. The COstatus system measures cardiac output using ultrasound dilution technology. This device has been validated in a pig model against thermodilution, which is considered the gold standard for measuring cardiac output [25]. The COstatus was used for measurements of cardiac output (to confirm that the pigs achieved the septic shock injury criteria) and CBVI, which is the volume of blood in the heart and lungs indexed to weight in kg. Baseline measurements of heart rate, blood pressure, cardiac output, and CBVI were obtained.

### Experimental design

**Fig. 1:**
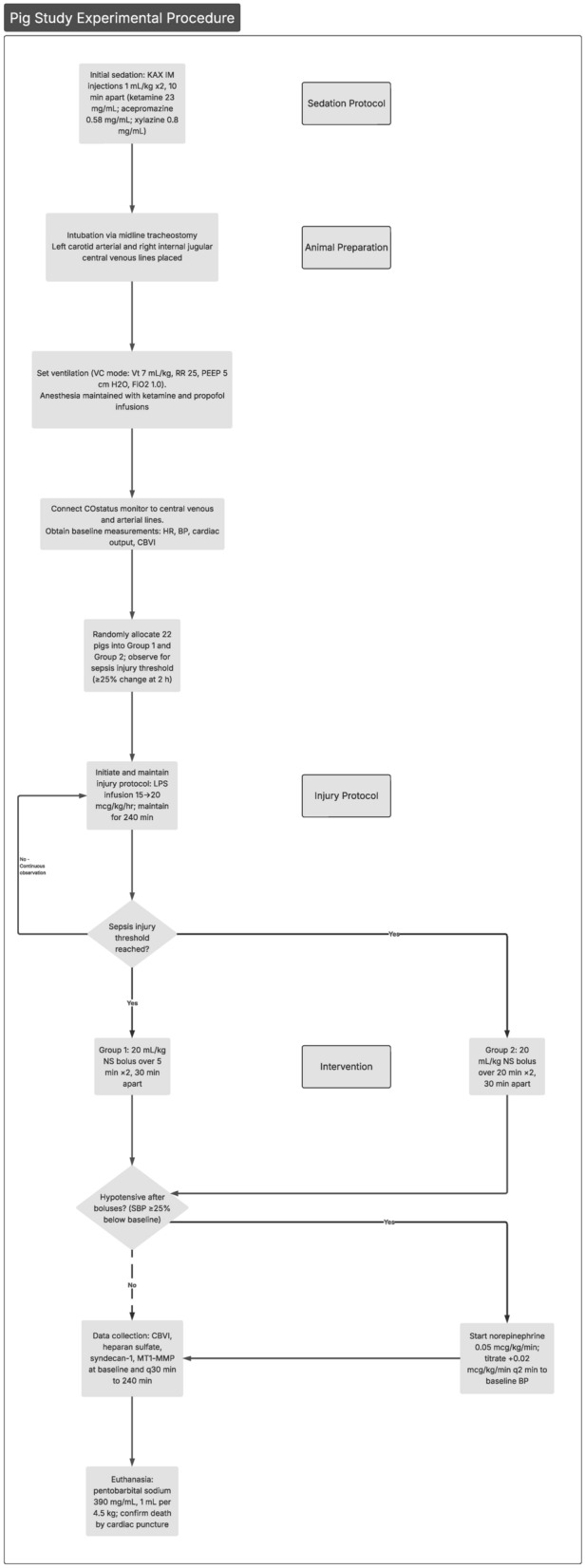
Experimental protocol. Pigs were sedated, intubated via tracheostomy, and instrumented with arterial and central venous lines for hemodynamic monitoring. After baseline measurements, endotoxemia was induced with a continuous lipopolysaccharide infusion for up to 240 min. Animals were randomized to receive two normal saline boluses (20 mL/kg) administered either rapidly (over 5 min) or slowly (over 20 min), separated by 30 min, once predefined sepsis injury thresholds were reached. Hypotension after the two initial fluid boluses (≥ 25% decrease in systolic blood pressure from baseline) was treated with titrated norepinephrine. Hemodynamic variables and biomarkers were collected at baseline and every 30 min until study completion, followed by euthanasia

Injury protocol (Fig. 1): After a 30-min stabilization period, the piglets from both groups received an intravenous infusion of lipopolysaccharide from *Escherichia coli* serotype 0111:B4, run as a continuous infusion. The infusion was started at a dose of 15 mcg/kg/h and increased by 1 mcg/kg/h every 10 min to a maximum dose of 20 mcg/kg/h. The infusion was then maintained at this rate for the remainder of the study (total = 240 min).

On each laboratory day, we randomly allocated the 22 pigs in the study into two groups using a paper lottery system. After initiating the injury protocol, the pigs were observed until they reached the sepsis injury threshold. The septic shock injury threshold was defined by the occurrence of any of the following: a ≥ 25% decrease in systolic blood pressure at any time point, a ≥ 25% increase in heart rate at 2 h, or a ≥ 25% decrease in cardiac index or a ≥ 25% increase in systemic vascular resistance index from baseline at 2 h. These cutoffs were based on prior experience with lipopolysaccharide in a pig model to ensure clinically relevant hemodynamic compromise while preserving survival to the protocol end given that lipopolysaccharide induces severe hemodynamic instability. If the blood pressure target was achieved before the 2-h time point, that moment was designated as the onset of septic shock, as inflammatory injury progresses rapidly once hypotension develops. Other criteria, which reflect earlier physiologic changes, were assessed at the 2-h mark, as this time point generally corresponds to activation of the inflammatory cascade.

After reaching the septic shock injury threshold, the pigs received a 20 mL/kg bolus of 0.9% normal saline. The pigs in group 1 received the bolus over 5 min, and the pigs in group 2 received it over 20 min. Each group received two fluid boluses 30 min apart from the start of the fluid bolus initiation. The time of administration of fluids was based on a prior study that defined a fast bolus as over 5–10 min and a slow bolus over 15–20 min, demonstrating a greater need for intubation in those patients who received the bolus as a fast bolus, compared with slower administration of the fluids [11]. If at any point in the study protocol after administration of the fluid boluses the animals remained hypotensive (systolic blood pressure 25% below baseline), norepinephrine was administered to maintain blood pressure, with the norepinephrine starting at 0.05 mcg/kg/min and titrated by 0.02 mcg/kg/min every 2 min to achieve the pig’s baseline (preinjury) blood pressure. No additional fluid boluses were administered to ensure that the quantity of fluid received by both groups was the same.

Data collection: CBVI, plasma heparan sulfate, syndecan-1, and MT1-MMP levels were obtained at injury baseline (onset of septic shock) and every 30 min thereafter until the completion of the 240-min study protocol. The sample size was calculated based on the change in central blood volume. The mean baseline central blood volume in pigs this age is 407 mL (SD = 89) (unpublished data from previous studies in this laboratory). Assuming that the central blood volume in the fast FBT group will be 25% less than that in the slow FBT group due to glycocalyx degradation, and assuming an alpha of 0.05, beta of 0.2, and power of 0.8, 12 pigs were needed in each group to demonstrate this difference. The study was stopped after 11 pigs in each group, as it was felt that an additional pig in each group would not change the outcome of the study. Therefore, to be judicious with the unnecessary use of animals, the study was stopped after 11 animals in each group.

Euthanasia protocol: Following completion of the experimental protocol, the pigs were administered an injection of 390 mg/ml pentobarbital sodium, 1 ml for each 4.5 kg body weight. The death of the animal was confirmed using cardiac puncture. If a heartbeat was still present after the cardiac puncture, an additional dose of pentobarbital sodium was administered, and the cardiac puncture was repeated.

Statistical analysis: Injury time and postinjury data were summarized by flow type and half-hour intervals for 2 h. A linear mixed-effects model with repeated-measures analysis of variance was used to compare the mean changes over time postinjury between the fast and slow flow groups. The model was adjusted for baseline values of the corresponding outcome variable, and a within-animal first-order autoregressive correlation structure was assumed. Flow group, time since injury, their interaction, and baseline values were included as independent variables in the model. Pairwise comparisons and changes from baseline were generated as needed. Model assumptions were checked prior to analysis. All tests were two-tailed with a significance level of 0.05. The statistical software R version 4.5.2 was used for data analysis [26].

## Results

**Fig. 2:**
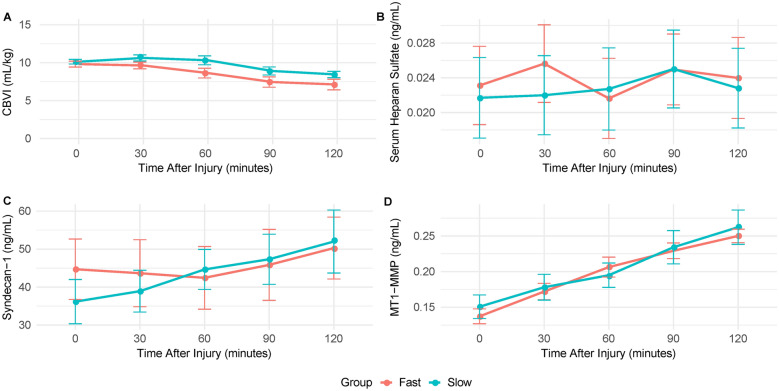
Temporal changes in biomarkers of endothelial glycocalyx shedding and central blood volume index following onset of septic shock. **A** Central blood volume index measured over time after septic shock onset. **B**–**D** Circulating markers of glycocalyx degradation, including heparan sulfate (**B**), syndecan-1 (**C**), and membrane type-1 matrix metalloproteinase (MT1-MMP) (**D**), measured at serial time points following onset of septic shock. Data are shown for animals stratified into fast and slow intravenous fluid groups

Table 1 describes the basic characteristics of the animals in each group. Figure 2 demonstrates the trends of CBVI, plasma heparan sulfate, syndecan-1, and MT1-MMP from the onset of septic shock through 2 h after septic shock onset (five measurements from minutes 120 through 240 of the study). Table 2 summarizes the mean (changes) over time by the fast IVF and slow IVF groups throughout the study, and Table 3 summarizes the mean changes from septic shock onset to the last measurement (120 min) within each group. There was no significant between-group difference in the overall mean plasma level of heparan sulfate and syndecan-1 between the fast IVF group and the slow IVF group over the study (*P* = 0.405 and 0.571, respectively). MT1-MMP levels increased in both groups as an effect of time, as demonstrated in Table 3. However, there was no difference in the overall mean between the groups over time (*P* = 0.737). The baseline-adjusted mean CBVI over the four postintervention measurements was greater in the slow fluid group compared with the fast fluid group; however, the overall mean difference fell short of achieving statistical significance (*P* = 0.073), indicating a nonsignificant trend toward a greater CBVI with slower infusion. The mean CBVI declined significantly from septic shock onset to the last measurement (120 min) in both groups (fast: −2.62 mL/kg, *P* < 0.001; slow: −1.83 mL/kg, *P* = 0.003). Of note, of the 22 animals in the study, five pigs (two in the slow IVF group and three in the fast IVF group) did not have a final CBVI reading, due to inadequate cardiac output.

**Table 1:** Basic characteristics of the animals in each group

Variables	Fast	Slow	*P* value
	(*N* = 11)	(*N* = 11)	
Injury criteria, *n* (%)			
Blood pressure	2 (18.2)	5 (45.5)	0.381
Cardiac index	5 (45.5)	3 (27.3)	
Heart rate	4 (36.4)	3 (27.3)	
Norepinephrine, *n* (%)			
No	4 (36.4)	5 (45.5)	> 0.99
Yes	7 (63.6)	6 (54.5)	
Time until norepinephrine in minutes			
Mean (SEM)	200 (13)	180 (5.5)	0.27
Median [Q1, Q2]	200 [180, 210]	180 [180, 180]	
Sex, *n* (%)			
Female	4 (36.4)	5 (45.5)	> 0.99
Male	7 (63.6)	6 (54.5)	
Weight			
Mean (SEM)	30 (0.61)	30 (0.54)	0.733
Median [Q1, Q2]	31 [30, 32]	30 [30, 32]	
Time to injury in minutes			
Mean (SEM)	130 (5.6)	120 (3.9)	0.248
Median [Q1, Q2]	130 [120, 130]	120 [110, 130]	
Missing	0 (0%)	1 (9.1%)	

*Q1* first quartile, *Q2* second quartile, *SEM* standard error of mean

**Table 2:** Postinjury biomarker mean (standard error) by flow type

Variables	Summary	Postinjury time										*P value
		0 min		30 min		60 min		90 min		120 min		
		Fast	Slow	Fast	Slow	Fast	Slow	Fast	Slow	Fast	Slow	
CBVI	Mean (SEM)	9.8 (0.39)	10 (0.31)	9.6 (0.44)	11 (0.41)	8.6 (0.64)	10 (0.59)	7.5 (0.68)	8.9 (0.53)	7.1 (0.82)	8.4 (0.46)	0.073
HS	Mean (SEM)	0.023 (0.005)	0.022 (0.005)	0.026 (0.005)	0.022 (0.005)	0.022 (0.005)	0.023 (0.005)	0.025 (0.004)	0.025 (0.005)	0.024 (0.005)	0.023 (0.005)	0.405
SDC_1	Mean (SEM)	45 (8.4)	36 (5.8)	44 (8.8)	39 (5.5)	42 (8.2)	45 (5.3)	46 (9.3)	47 (6.6)	50 (8.1)	52 (8.3)	0.571
MT1-MMP	Mean (SEM)	0.14 (0.011)	0.15 (0.017)	0.17 (0.012)	0.18 (0.018)	0.21 (0.014)	0.19 (0.017)	0.23 (0.011)	0.23 (0.023)	0.25 (0.009)	0.26 (0.024)	0.737

*CBVI* central blood volume index, *HS* heparan sulfate, *MT1-MMP* membrane type-1 matrix metalloproteinase, *SDC_1* syndecan-1, *SEM* standard error of mean

^*^The *P* value to compare the overall postinjury mean difference between fast and slow flow after adjustment for baseline

**Table 3:** Mean change from baseline to 120 min postinjury time

	Mean difference from baseline to 120 min postinjury time within each group			
	Fast IVF		Slow IVF	
	Mean (SE)	*P* value	Mean (SE)	*P* value
CBVI	−2.62 (0.54)	< 0.001	−1.83 (0.51)	0.003
HS	0.0009 (0.0014)	0.521	0.0011 (0.0014)	0.435
SDC_1	11.91 (7.68)	0.138	13.83 (7.32)	0.075
MT1-MMP	0.11 (0.01)	< 0.001	0.11 (0.01)	< 0.001

*CBVI* central blood volume index, *HS* heparan sulfate, *IVF* intravenous fluid, *MT1-MMP* membrane type-1 matrix metalloproteinase, *SDC_1* syndecan-1, *SE* standard error

Post hoc comparisons at each individual time point revealed no significant differences between the fast flow and slow flow groups for heparan sulfate, syndecan-1, or MT1-MMP, while CBVI showed a consistent but nonsignificant trend toward divergence between groups.

## Discussion

This study aimed to evaluate the effect of fast versus slow administration of IVF on the glycocalyx by measuring the plasma concentration of heparan sulfate and syndecan-1 and the intervascular fluid volume by measuring the central blood volume index in a porcine septic shock model. These biomarkers are well-established breakdown products of the glycocalyx, and elevated levels correlate with a more significant degree of glycocalyx breakdown and injury [20, 21]. MT1-MMP levels were also compared between the fast and slow IVF groups to determine whether faster administration of IVF results in shearing injury to the vascular endothelium. MT1-MMP level increases with increased shearing forces on the glycocalyx [22]. The CBVI and the three biomarkers were measured at the time of sepsis onset and every 30 min after the administration of IVF for a total of 120 min.

There was no significant difference in the glycocalyx breakdown products, heparan sulfate and syndecan-1 between the two groups, indicating that the glycocalyx breakdown between the fast and slow IVF groups was not statistically different. Although there was a significant increase in the MT1-MMP levels as the septic shock progressed, there was no significant difference in MT1-MMP levels between the two groups, suggesting that the fast IVF group did not result in increased shearing forces on the vascular endothelium compared with the slow IVF group. The increase in MT1-MMP levels over time observed in both groups is likely a result of the inflammatory state induced by lipopolysaccharide endotoxemia. TNF-α and interleukin-1 have both been shown to increase MT1-MMP levels [27]. Syndecan-1 is well established as a sensitive marker of septic shock and typically rises during its course. In this study, syndecan-1 levels did not increase significantly over the 2 h that it was measured, likely because this window was insufficient to capture its elevation. Most studies evaluating syndecan-1 kinetics measure levels several hours to days after sepsis onset, rather than this early time point. Therefore, any observed change in syndecan-1 in our study would more likely reflect the effects of intravenous fluid administration rather than the septic shock itself [28, 29].

There was a difference in CBVI between the fast and slow IVF groups, with the slow-infusion group showing higher CBVI. Although this difference did not reach statistical significance, likely due to the limited sample size, the trend favored greater intravascular volume with slower infusion rates. Because markers of glycocalyx injury and vascular stress were similar between groups, the lower CBVI in the fast-infusion group is most plausibly explained by increased hydrostatic pressure driving fluid out of the vascular space. This finding is crucial, as it suggests that slower IVF bolus administration in septic shock may promote better intravascular fluid retention compared with rapid infusion [28, 29].

FBT is a cornerstone in treating patients with septic shock. The role of FBT is to improve circulating blood volume, cardiac output, and oxygen delivery, as well as to mitigate circulatory dysfunction and organ hypoperfusion caused by vasodilation and capillary leak syndrome resulting from sepsis [30]. Used correctly, FBT in sepsis and septic shock patients has been shown to decrease heart rate, improve systolic blood pressure, mean arterial pressure, central venous pressure, stroke volume, cardiac index, and systemic vascular resistance [31].

There are detrimental effects of FBT, including critical fluid overload, electrolyte derangements, cardiac dysfunction, and respiratory failure [31, 32]. The benefit that patients receive from FBT is highly transient. Numerous studies have demonstrated that the hemodynamic benefit seen from FBT will remain for approximately 2 h [7, 10, 33–36]. This effect is due to extravasation of the fluid from the intravascular compartment to the extravascular space. Among septic patients, whose vasculature integrity is compromised, less than 5% of a fluid bolus remains in the intravascular space after 1 h [9].

The glycocalyx is the endothelial layer responsible for regulating vascular permeability and microvascular tone. It is composed of proteoglycans, glycoproteins bound with sialic acid, glycosaminoglycans, and associated plasma proteins [37, 38]. Sepsis degrades the glycocalyx, leading to loss of vascular integrity. Inflammatory cytokines, especially TNF-α, have been implicated in the degradation of the glycocalyx in sepsis [16–19]. However, damage to the glycocalyx from the inflammatory mediators is exacerbated by a second hit, with studies demonstrating that IVF can damage the glycocalyx [13–15, 20, 21]. Although the exact mechanism of how IVF can worsen glycocalyx degradation in septic shock has been elusive, several theories have been proposed, including inflammatory effects of IVF on the glycocalyx [39–41], damage to the glycocalyx through stretching forces on the vasculature from volume overload [42–44], or secondary to the effects of shearing forces on the glycocalyx [20, 21]. Prior studies have shown that larger volumes of IVF and unbalanced IVF solutions (0.9% normal saline compared with balanced solutions, such as Plasma-Lyte [Baxter, Deerfield, IL] or lactated Ringers) are associated with more significant damage to the glycocalyx [14, 20, 21]. It has been speculated, although not proven, that the speed of administration of IVF can also play a role in the degree of damage induced by the IVF to the glycocalyx, with faster administration of the fluids resulting in more severe glycocalyx degradation compared with slower administration of IVF. One suspected mechanism for the worsening effect of fast IVF administration on the glycocalyx is the shearing forces exerted by the fluids on the delicate endothelial lining [20].

Thus, the results of this study add to the current body of literature on the glycocalyx in sepsis, demonstrating that the speed of IVF administration did not worsen the glycocalyx breakdown compared with slower IVF delivery. However, independent of the effects of the IVF on the glycocalyx, slower IVF administration was associated with a greater degree of intravascular retention of the IVF compared with faster IVF administration.

Although there are studies demonstrating a lack of an effect of IVF on endothelial glycocalyx breakdown, this study adds context to the existing literature in several ways. The studies by MacDonald et al. and Oshima et al. both demonstrated that there was no relationship between fluid volume and the degree of endothelial breakdown [45, 46]. However, both studies measured glycocalyx breakdown products only at enrollment and 24 h later. Thus, the serum levels of heparan sulfate and syndecan-1 may not exclusively reflect the effect of IVF on the glycocalyx, given that over the 24-h period as sepsis was evolving, several factors, including cytokines, worsen glycocalyx breakdown. Sepsis is known to enhance the induction of glucuronidase heparanase, which cleaves glycocalyx-bound heparan sulfates, thereby releasing its fragments into the circulation [47]. Heparanase also degrades underlying proteoglycans, such as syndecan-1. Loss of the heparan sulfate branches exposes the underlying protein core to inflammatory proteases, leading to protease-mediated syndecan-1 shedding [45–47]. Sepsis itself may also upregulate endothelial syndecan-1 expression, which may also affect the circulating concentration seen 24 h into sepsis [48]. Given that most studies examine glycocalyx breakdown products in sepsis at the 24-h mark, and that at that point, multiple variables have affected glycocalyx breakdown, it is hard to fully appreciate or isolate the effect of fluids on the glycocalyx, and the effect of IVF may be overshadowed by the effects of the inflammatory cascade on glycocalyx breakdown.

Conversely, this current study examined glycocalyx breakdown with the onset of septic shock, immediately after fluid administration, and serially every half hour for 2 h, stopping 2 h after the onset of sepsis. It is unlikely that in that short time frame the inflammatory effect of sepsis would be able to have an appreciable effect on the glycocalyx. Therefore, any observed change could be attributed primarily to the effect of IVF. One additional strength of this study is that it demonstrates worsening vascular permeability, as reflected by lower central blood volumes, in the faster IVF group compared with the slower IVF group.

This study has several limitations. First, as with all animal models of sepsis, the translational applicability to human septic shock is uncertain. The lipopolysaccharide-induced septic shock model, while allowing for standardized injury induction and controlled experimental conditions, does not fully replicate the heterogeneous and complex pathophysiology of human sepsis, with the variable immune responses and diverse patient comorbidities. The lipopolysaccharide model creates a more homogeneous inflammatory response than what occurs clinically, potentially limiting the generalizability of our findings to the varied presentations of sepsis in human patients. Second, the relatively short duration of the study protocol (240 min total observation) may not capture the longer-term consequences of fluid bolus therapy on glycocalyx integrity and organ dysfunction that develop over hours to days in clinical sepsis. While our time course allowed assessment of acute glycocalyx shedding and hemodynamic changes, delayed effects on endothelial recovery, capillary leak progression, and organ injury may have been missed. Third, the sample size (*n* = 22 total, 11 per group) was relatively small and may have been inadequate to detect statistically significant differences in some outcomes, particularly the CBVI, where a trend toward significance (*P* = 0.073) was observed, and would likely have reached statistical significance with a larger sample size. Fourth, the septic shock criteria used in this study differed from the traditional Sepsis-3 definition because LPS endotoxin induces a rapid and profound inflammatory response. Waiting for full Sepsis-3 criteria to be met would likely have resulted in animal mortality before completion of the study period. The criteria selected to initiate therapy reflect clear hemodynamic deterioration and onset of systemic inflammation. Thus, while these criteria may not technically fulfill Sepsis-3 definitions, they capture a clinically meaningful inflammatory state relevant to the study objectives. Finally, the study used 0.9% normal saline as the resuscitation fluid, which is known to be more harmful to the glycocalyx than balanced crystalloid solutions. While this choice was intentional to maximize potential differences in glycocalyx injury between groups, the use of only one fluid type limits our ability to generalize findings to other resuscitation fluids that may have different effects on the endothelium.

Despite these limitations, this porcine model provided a controlled environment for investigating the mechanistic effects of fluid bolus administration rate on endothelial glycocalyx injury in septic shock, generating hypothesis-forming data that warrant further investigation in larger, more heterogeneous animal studies and ultimately in clinical trials.

## Conclusion

In this porcine model of septic shock using lipopolysaccharide endotoxin, based on plasma levels of syndecan-1 and heparan sulfate, there does not appear to be a difference in glycocalyx degradation between the cohort that received fast IVF and the cohort that received slow IVF. Shear stress also did not differ between the two groups, as measured by plasma MT1-MMP concentrations. However, the CBVI trended higher in the group that received slow IVF, potentially indicating an advantage to slower delivery of an IVF bolus in terms of a greater volume remaining in the vascular space.

## Data Availability

The datasets used and/or analyzed during the current study are available from the corresponding author on reasonable request.

## Abbreviations

CBVI: Central blood volume index
FBT: Fluid bolus therapy
IVF: Intravenous fluid
MTI-MMP: Membrane type-1 matrix metalloproteinase
TNF-α: Tumor necrosis factor-alpha

## References

[1] Shankar-Hari M, Phillips GS, Levy ML, Seymour CW, Liu VX, Deutschman CS, Angus DC, Rubenfeld GD, Singer M, Sepsis Definitions Task Force (2016) Developing a new definition and assessing new clinical criteria for septic shock: for the Third International Consensus Definitions for Sepsis and Septic Shock (Sepsis-3). JAMA 315:775–787 26903336 10.1001/jama.2016.0289 PMC4910392

[2] GBD 2021 Global Sepsis Collaborators (2025) Global, regional, and national sepsis incidence and mortality, 1990-2021: a systematic analysis. Lancet Glob Health 13:e2013–e2026 41135560 10.1016/S2214-109X(25)00356-0

[3] Schlapbach LJ, Straney L, Alexander J, MacLaren G, Festa M, Schibler A, Slater A, ANZICS Paediatric Study Group (2015) Mortality related to invasive infections, sepsis, and septic shock in critically ill children in Australia and New Zealand, 2002-13: a multicentre retrospective cohort study. Lancet Infect Dis 15:46–54 25471555 10.1016/S1473-3099(14)71003-5

[4] Weiss SL, Balamuth F, Chilutti M, Ramos MJ, McBride P, Kelly NA, Payton KJ, Fitzgerald JC, Pennington JW (2020) Identification of pediatric sepsis for epidemiologic surveillance using electronic clinical data. Pediatr Crit Care Med 21:113–121 32032262 10.1097/PCC.0000000000002170 PMC7008717

[5] Fleischmann-Struzek C, Goldfarb DM, Schlattmann P, Schlapbach LJ, Reinhart K, Kissoon N (2018) The global burden of paediatric and neonatal sepsis: a systematic review. Lancet Respir Med 6:223–230 29508706 10.1016/S2213-2600(18)30063-8

[6] Weiss SL, Peters MJ, Alhazzani W, Agus MSD, Flori HR, Inwald DP, Nadel S, Schlapbach LJ, Tasker RC, Argent AC, Brierley J, Carcillo J, Carrol ED, Carroll CL, Cheifetz IM, Choong K, Cies JJ, Cruz AT, De Luca D, Deep A, Faust SN, De Oliveira CF, Hall MW, Ishimine P, Javouhey E, Joosten KFM, Joshi P, Karam O, Kneyber MCJ, Lemson J, MacLaren G, Mehta NM, Møller MH, Newth CJL, Nguyen TC, Nishisaki A, Nunnally ME, Parker MM, Paul RM, Randolph AG, Ranjit S, Romer LH, Scott HF, Tume LN, Verger JT, Williams EA, Wolf J, Wong HR, Zimmerman JJ, Kissoon N, Tissieres P (2020) Surviving Sepsis Campaign international guidelines for the management of septic shock and sepsis-associated organ dysfunction in children. Pediatr Crit Care Med 21:e52–e106 32032273 10.1097/PCC.0000000000002198

[7] Long E, Babl FE, Oakley E, Sheridan B, Duke T, Pediatric Research in Emergency Departments International Collaborative (2018) Cardiac index changes with fluid bolus therapy in children with sepsis–an observational study. Pediatr Crit Care Med 19:513–518 29533353 10.1097/PCC.0000000000001534

[8] McIlroy DR, Kharasch ED (2003) Acute intravascular volume expansion with rapidly administered crystalloid or colloid in the setting of moderate hypovolemia. Anesth Analg 96:1572–1577 12760977 10.1213/01.ANE.0000061460.59320.B0

[9] Sánchez M, Jiménez-Lendínez M, Cidoncha M, Asensio MJ, Herrerot E, Collado A, Santacruz M (2011) Comparison of fluid compartments and fluid responsiveness in septic and non-septic patients. Anaesth Intensive Care 39:1022–1029 22165353 10.1177/0310057X1103900607

[10] Nunes TSO, Ladeira RT, Bafi AT, de Azevedo LCP, Machado FR, Freitas FGR (2014) Duration of hemodynamic effects of crystalloids in patients with circulatory shock after initial resuscitation. Ann Intensive Care 4:25 25593742 10.1186/s13613-014-0025-9 PMC4273721

[11] Sankar J, Ismail J, Sankar MJ, S CP, Meena RS (2017) Fluid bolus over 15-20 versus 5-10 minutes each in the first hour of resuscitation in children with septic shock: a randomized controlled trial. Pediatr Crit Care Med 18:e435–e445 28777139 10.1097/PCC.0000000000001269

[12] Saoraya J, Wongsamita L, Srisawat N, Musikatavorn K (2021) The effects of a limited infusion rate of fluid in the early resuscitation of sepsis on glycocalyx shedding measured by plasma syndecan-1: a randomized controlled trial. J Intensive Care 9:1 33402229 10.1186/s40560-020-00515-7 PMC7784279

[13] Fernández-Sarmiento J, Molina CF, Salazar-Peláez LM, Flórez S, Alarcón-Forero LC, Sarta M, Hernández-Sarmiento R, Villar JC (2023) Biomarkers of glycocalyx injury and endothelial activation are associated with clinical outcomes in patients with sepsis: a systematic review and meta-analysis. J Intensive Care Med 38:95–105 35722738 10.1177/08850666221109186

[14] Fernández-Sarmiento J, Salazar-Peláez LM, Acevedo L, Niño-Serna LF, Flórez S, Alarcón-Forero L, Mulett H, Gómez L, Villar JC (2023) Endothelial and glycocalyx biomarkers in children with sepsis after one bolus of unbalanced or balanced crystalloids. Pediatr Crit Care Med 24:213–221 36598246 10.1097/PCC.0000000000003123

[15] Fernández-Sarmiento J, Salazar-Peláez LM, Carcillo JA (2020) The endothelial glycocalyx: a fundamental determinant of vascular permeability in sepsis. Pediatr Crit Care Med 21:e291–e300 32132499 10.1097/PCC.0000000000002266 PMC9084566

[16] Chappell D, Hofmann-Kiefer K, Jacob M, Rehm M, Briegel J, Welsch U, Conzen P, Becker BF (2009) TNF-alpha induced shedding of the endothelial glycocalyx is prevented by hydrocortisone and antithrombin. Basic Res Cardiol 104:78–89 18836678 10.1007/s00395-008-0749-5

[17] Nelson A, Berkestedt I, Bodelsson M (2014) Circulating glycosaminoglycan species in septic shock. Acta Anaesthesiol Scand 58:36–43 24341693 10.1111/aas.12223

[18] Nieuwdorp M, Meuwese MC, Mooij HL, van Lieshout MHP, Hayden A, Levi M, Meijers JCM, Ince C, Kastelein JJP, Vink H, Stroes ESG (2009) Tumor necrosis factor-alpha inhibition protects against endotoxin-induced endothelial glycocalyx perturbation. Atherosclerosis 202:296–303 18550063 10.1016/j.atherosclerosis.2008.03.024

[19] Wiesinger A, Peters W, Chappell D, Kentrup D, Reuter S, Pavenstädt H, Oberleithner H, Kümpers P (2013) Nanomechanics of the endothelial glycocalyx in experimental sepsis. PLoS ONE 8:e80905 24278345 10.1371/journal.pone.0080905 PMC3835794

[20] Hippensteel JA, Uchimido R, Tyler PD, Burke RC, Han X, Zhang F, McMurtry SA, Colbert JF, Lindsell CJ, Angus DC, Kellum JA, Yealy DM, Linhardt RJ, Shapiro NI, Schmidt EP (2019) Intravenous fluid resuscitation is associated with septic endothelial glycocalyx degradation. Crit Care 23:259 31337421 10.1186/s13054-019-2534-2 PMC6652002

[21] Powell MF, Mathru M, Brandon A, Patel R, Frölich MA (2014) Assessment of endothelial glycocalyx disruption in term parturients receiving a fluid bolus before spinal anesthesia: a prospective observational study. Int J Obstet Anesth 23:330–334 25201316 10.1016/j.ijoa.2014.06.001

[22] Kang H, Duran CL, Abbey CA, Kaunas RR, Bayless KJ (2015) Fluid shear stress promotes proprotein convertase-dependent activation of MT1-MMP. Biochem Biophys Res Commun 460:596–602 25800869 10.1016/j.bbrc.2015.03.075 PMC4428763

[23] Lutz J, Levenbrown Y, Hossain MJ, Hesek A, Massa KE, Keith JP, Shaffer TH (2023) Impact of intravenous fluid administration on cardiac output and oxygenation during cardiopulmonary resuscitation. Intensive Care Med Exp 11:13 36959337 10.1186/s40635-023-00497-4 PMC10036707

[24] Levenbrown Y, Hossain MJ, Keith JP, Burr K, Hesek A, Shaffer T (2020) The effect of positive end-expiratory pressure on cardiac output and oxygen delivery during cardiopulmonary resuscitation. Intensive Care Med Exp 8:36 32712733 10.1186/s40635-020-00330-2 PMC7382317

[25] Darling E, Thuramalla N, Searles B (2011) Validation of cardiac output measurement by ultrasound dilution technique with pulmonary artery thermodilution in a pediatric animal model. Pediatr Cardiol 32:585–589 21359950 10.1007/s00246-011-9915-x PMC3108493

[26] R Core Team (2021) R: A language and enviroment for statistical computing. R Foundation for Statistical Computing, Vienna, Austria. https://www.R-project.org/

[27] Rajavashisth TB, Xu XP, Jovinge S, Meisel S, Xu XO, Chai NN, Fishbein MC, Kaul S, Cercek B, Sharifi B, Shah PK (1999) Membrane type 1 matrix metalloproteinase expression in human atherosclerotic plaques: evidence for activation by proinflammatory mediators. Circulation 99:3103–3109 10377072 10.1161/01.cir.99.24.3103

[28] Kajita Y, Terashima T, Mori H, Islam MM, Irahara T, Tsuda M, Kano H, Takeyama N (2021) A longitudinal change of syndecan-1 predicts risk of acute respiratory distress syndrome and cumulative fluid balance in patients with septic shock: a preliminary study. J Intensive Care 9:27 33726863 10.1186/s40560-021-00543-x PMC7962080

[29] Huang X, Hu H, Sun T, Zhu W, Tian H, Hao D, Wang T, Wang X (2021) Plasma endothelial glycocalyx components as a potential biomarker for predicting the development of disseminated intravascular coagulation in patients with sepsis. J Intensive Care Med 36:1286–1295 32799720 10.1177/0885066620949131

[30] Gelbart B (2018) Fluid bolus therapy in pediatric sepsis: current knowledge and future direction. Front Pediatr 6:308 30410875 10.3389/fped.2018.00308 PMC6209667

[31] Selewski DT, Gist KM, Nathan AT, Goldstein SL, Boohaker LJ, Akcan-Arikan A, Bonachea EM, Hanna M, Joseph C, Mahan JD, Mammen C, Nada A, Reidy K, Staples A, Wintermark P, Griffin R, Askenazi DJ, Guillet R, Neonatal Kidney Collaborative (2020) The impact of fluid balance on outcomes in premature neonates: a report from the AWAKEN study group. Pediatr Res 87:550–557 31537009 10.1038/s41390-019-0579-1 PMC7036003

[32] Loomba RS, Villarreal EG, Farias JS, Flores S, Bronicki RA (2022) Fluid bolus administration in children, who responds and how? A systematic review and meta-analysis. Paediatr Anaesth 32:993–999 35736026 10.1111/pan.14512

[33] Becher T, Wendler A, Eimer C, Weiler N, Frerichs I (2019) Changes in electrical impedance tomography findings of ICU patients during rapid infusion of a fluid bolus: a prospective observational study. Am J Respir Crit Care Med 199:1572–1575 30875244 10.1164/rccm.201812-2252LE

[34] Bihari S, Prakash S, Bersten AD (2013) Post resusicitation fluid boluses in severe sepsis or septic shock: prevalence and efficacy (price study). Shock 40:28–34 23635850 10.1097/SHK.0b013e31829727f1

[35] Ke L, Calzavacca P, Bailey M, May CN, Li WQ, Bertolini J, Bellomo R (2014) Systemic and renal haemodynamic effects of fluid bolus therapy: sodium chloride versus sodium octanoate-balanced solution. Crit Care Resusc 16:29–33 24588433

[36] Lankadeva YR, Kosaka J, Iguchi N, Evans RG, Booth LC, Bellomo R, May CN (2019) Effects of fluid bolus therapy on renal perfusion, oxygenation, and function in early experimental septic kidney injury. Crit Care Med 47:e36–e43 30394921 10.1097/CCM.0000000000003507

[37] Reitsma S, Slaaf DW, Vink H, van Zandvoort MAMJ, Oude Egbrink MGA (2007) The endothelial glycocalyx: composition, functions, and visualization. Pflugers Arch 454:345–359 17256154 10.1007/s00424-007-0212-8 PMC1915585

[38] Uchimido R, Schmidt EP, Shapiro NI (2019) The glycocalyx: a novel diagnostic and therapeutic target in sepsis. Crit Care 23:16 30654825 10.1186/s13054-018-2292-6 PMC6337861

[39] Rhee P, Wang D, Ruff P, Austin B, DeBraux S, Wolcott K, Burris D, Ling G, Sun L (2000) Human neutrophil activation and increased adhesion by various resuscitation fluids. Crit Care Med 28:74–78 10667502 10.1097/00003246-200001000-00012

[40] Suzuki K, Okada H, Takemura G, Takada C, Kuroda A, Yano H, Zaikokuji R, Morishita K, Tomita H, Oda K, Matsuo S, Uchida A, Fukuta T, Sampei S, Miyazaki N, Kawaguchi T, Watanabe T, Yoshida T, Ushikoshi H, Yoshida S, Maekawa Y, Ogura S (2019) Neutrophil elastase damages the pulmonary endothelial glycocalyx in lipopolysaccharide-induced experimental endotoxemia. Am J Pathol 189:1526–1535 31108101 10.1016/j.ajpath.2019.05.002

[41] van Haren FMP, Sleigh J, Cursons R, La Pine M, Pickkers P, van der Hoeven JG (2011) The effects of hypertonic fluid administration on the gene expression of inflammatory mediators in circulating leucocytes in patients with septic shock: a preliminary study. Ann Intensive Care 1:44 22044529 10.1186/2110-5820-1-44 PMC3217886

[42] Bruegger D, Jacob M, Rehm M, Loetsch M, Welsch U, Conzen P, Becker BF (2005) Atrial natriuretic peptide induces shedding of endothelial glycocalyx in coronary vascular bed of guinea pig hearts. Am J Physiol Heart Circ Physiol 289:H1993–H1999 15964925 10.1152/ajpheart.00218.2005

[43] Chappell D, Bruegger D, Potzel J, Jacob M, Brettner F, Vogeser M, Conzen P, Becker BF, Rehm M (2014) Hypervolemia increases release of atrial natriuretic peptide and shedding of the endothelial glycocalyx. Crit Care 18:538 25497357 10.1186/s13054-014-0538-5 PMC4201669

[44] Jacob M, Saller T, Chappell D, Rehm M, Welsch U, Becker BF (2013) Physiological levels of A-, B- and C-type natriuretic peptide shed the endothelial glycocalyx and enhance vascular permeability. Basic Res Cardiol 108:347 23563917 10.1007/s00395-013-0347-z

[45] Macdonald S, Bosio E, Shapiro NI, Balmer L, Burrows S, Hibbs M, Jowitt T, Smart L, Arendts G, Fatovich D (2022) No association between intravenous fluid volume and endothelial glycocalyx shedding in patients undergoing resuscitation for sepsis in the emergency department. Sci Rep 12:8733 35610344 10.1038/s41598-022-12752-x PMC9130214

[46] Oshima K, Di Gravio C, Yan B, McMurtry SA, Burke R, Levoir LM, Kravitz MS, Stephenson D, Issaian A, Hansen KC, D’Alessandro A, Douglas IS, Self WH, Lindsell CJ, Schildcrout JS, Schmidt EP, Shapiro NI (2025) Endothelial glycocalyx degradation in sepsis: analysis of the crystalloid liberal or vasopressors early resuscitation in sepsis (CLOVERS) trial, a multicenter, phase 3, randomized trial. Ann Am Thorac Soc 22:1382–1393 40315385 10.1513/AnnalsATS.202501-012OC PMC12416157

[47] Schmidt EP, Yang Y, Janssen WJ, Gandjeva A, Perez MJ, Barthel L, Zemens RL, Bowman JC, Koyanagi DE, Yunt ZX, Smith LP, Cheng SS, Overdier KH, Thompson KR, Geraci MW, Douglas IS, Pearse DB, Tudor RM (2012) The pulmonary endothelial glycocalyx regulates neutrophil adhesion and lung injury during experimental sepsis. Nat Med 18:1217–1223 22820644 10.1038/nm.2843 PMC3723751

[48] Strand ME, Herum KM, Rana ZA, Skrbic B, Askevold ET, Dahl CP, Vistnes M, Hasic A, Kvaløy H, Sjaastad I, Carlson CR, Tønnessen T, Gullestad L, Christensen G, Lunde IG (2013) Innate immune signaling induces expression and shedding of the heparan sulfate proteoglycan syndecan-4 in cardiac fibroblasts and myocytes, affecting inflammation in the pressure-overloaded heart. FEBS J 280:2228–2247 23374111 10.1111/febs.12161

